# The Role of Multimodality Imaging in HIV-Associated Cardiomyopathy

**DOI:** 10.3389/fcvm.2021.811593

**Published:** 2022-01-26

**Authors:** Ellise T. Gambahaya, Rimsha Rana, Shashwatee Bagchi, Garima Sharma, Sudipa Sarkar, Erin Goerlich, Blanche Cupido, Monica Mukherjee, Allison G. Hays

**Affiliations:** ^1^Division of Cardiology, Department of Medicine, University of Cape Town and Groote Schuur Hospital, Observatory, Cape Town, South Africa; ^2^Department of Medicine, Georgetown University School of Medicine, Baltimore, MD, United States; ^3^Division of Infectious Disease and Institute of Human Virology, University of Maryland School of Medicine, Baltimore, MD, United States; ^4^Division of Cardiology, Department of Medicine, Johns Hopkins University School of Medicine, Baltimore, MD, United States; ^5^Ciccarone Center for the Prevention of Cardiovascular Disease, Johns Hopkins University School of Medicine, Baltimore, MD, United States; ^6^Division of Endocrinology, Department of Medicine, Johns Hopkins University School of Medicine, Baltimore, MD, United States

**Keywords:** human immunodeficiency virus, cardiomyopathy, echocardiography, cardiac magnetic resonance imaging, computed cardiac tomography

## Abstract

Despite marked advances in therapeutics, HIV infection remains a leading cause of morbidity and mortality worldwide. HIV infection is associated with cardiovascular complications including myocardial dysfunction. The description of HIV-associated cardiomyopathy (HIVAC) has evolved over time from a predominantly dilated cardiomyopathy with systolic dysfunction to one of subclinical diastolic dysfunction. Multimodality cardiovascular imaging plays an integral role in our understanding of the etiology and pathogenesis of HIVAC. Such imaging is also essential in the evaluation of individuals with chronic HIV disease who present with cardiac symptoms, especially of heart failure. In the present review, we will highlight current evidence for the role of multimodality imaging in establishing the diagnosis, etiology and pathophysiology of HIVAC as well as guiding treatment and assessing prognosis.

## Introduction

Human immunodeficiency virus (HIV) infection remains a leading cause of morbidity and mortality worldwide (1). Of the estimated 37.9 million people living with HIV (PWH) worldwide, 25.6 million live in sub-Saharan Africa (2). There are ~1.2 million PWH in the United States (3). With the widespread advent of antiretroviral therapy (ART), HIV infection has largely become a chronic manageable condition. This is especially true in developed countries where ART is readily accessible. However, in some parts of the developing world, ART is not readily available and HIV remains an untreated and underrecognized condition characterized by progression to AIDS (acquired immune deficiency syndrome) and death.

HIV infection is associated with various cardiovascular manifestations. In settings where ART is readily accessible, PWH are living longer but continue to have chronic low-grade inflammation despite immunosuppression (4). In addition, as patients live longer traditional comorbidities such as hypertension, dyslipidaemia and diabetes with concomitant HIV infection contribute to accelerated atherosclerosis leading to vascular disease including coronary artery disease (CAD) (5). In fact, CAD has become the leading cause of cardiovascular mortality and morbidity in PWH (6). However, in resource limited settings where ART is not readily available, pericardial disease and myocardial disease in the form of myocarditis and cardiomyopathy are the leading causes of cardiac disease in PWH (7).

The presentation of myocardial disease in PWH ranges from incidental asymptomatic findings on autopsy or cardiovascular imaging to symptomatic heart failure. Decades after its recognition, the etiology and pathophysiology of HIV-associated cardiomyopathy (HIVAC) remains a topic of intense speculation with no consensus criteria for its definition. Nevertheless, its description has evolved from the pre-HAART era, when HIVAC was characterized by a dilated cardiomyopathy with systolic dysfunction associated with end-stage HIV disease and a poor prognosis. Morbidity and mortality occurred largely in the context of myocarditis, opportunistic infections, toxicity from medications, nutritional disorders, autoimmune mechanisms and inflammation. In more recent times, subclinical diastolic dysfunction has become the hallmark of HIVAC in individuals with treated HIV infection (8).

Cardiac imaging plays an integral role in the assessment of PWH who present with cardiac symptoms and signs especially those of heart failure. In the current review, we will highlight evidence for the role of multimodality imaging in establishing the diagnosis, etiology, and pathophysiology of HIVAC as well as guiding treatment and assessing prognosis.

## Epidemiology and Pathogenesis of HIV Associated Cardiomyopathy

The epidemiology of HIVAC has changed over the course of time from its initial description characterized by left ventricular (LV) systolic dysfunction to its current description of varying levels of diastolic dysfunction. Data on the prevalence of HIVAC is largely from studies conducted in the United States and Europe despite the fact that more than two-thirds of PWH are found in Sub-Saharan Africa (9). With the advent of ART, contemporary studies show that the prevalence of diastolic dysfunction is relatively high, with lower prevalence of systolic dysfunction. In a recent metanalysis of 11 studies conducted in Europe and the United States which included 2,242 mildly symptomatic and asymptomatic patients with HIV, the prevalence of systolic dysfunction was 8.3% and diastolic dysfunction was 43.4% (10). The Heart of Soweto Study conducted in South Africa showed that out of 5,328 cases of newly diagnosed heart failure, 518 (9.7%) were HIV positive. Moreover, 148 patients (29%) had systolic dysfunction and 196 (38%) had both systolic and diastolic dysfunction regardless of whether they were symptomatic for heart failure (7).

The pathogenesis of HIVAC is unclear but is likely multifactorial. Prior to the widespread implementation of ART, systolic dysfunction was thought to be mainly due to direct viral invasion of the myocardium, with or without myocarditis by the HIV virus or secondary to opportunistic infections such as toxoplasmosis and cryptococcosis (11). However, with the advent of ART other mechanisms of myocardial involvement in HIV infection have emerged including CAD and drug toxicity (12). Other putative mechanisms include autoimmunity, nutritional deficiencies and inflammation.

## Echocardiography

Echocardiography remains the first line imaging modality in assessing myocardial function. It is readily available, cost effective and robust in detecting both systolic and diastolic dysfunction. Prior to HAART, HIVAC was recognized on transthoracic echocardiography (TTE) as systolic LV dysfunction with varying degrees of LV dilatation. However, with the onset of HAART, HIVAC is largely recognized on TTE as subclinical diastolic dysfunction.

A study by Hakim et al. based in Zimbabwe aimed to determine the prevalence and characteristics of myocardial dysfunction in acutely ill HIV positive patients admitted to hospital. Out of a total of 151 patients, 14 (9%) had a dilated cardiomyopathy, 33 (22%) had LV dysfunction and 9 (6%) had isolated right ventricular (RV) dysfunction (13). These results mirrored a similar study in the United Kingdom which showed a relatively high prevalence of myocardial dysfunction of 26/173 (15%) patients (14). Dilated cardiomyopathy was a feature of advanced HIV disease and mean CD4 count was 38 cells/mm^3^ in these patients. The HIV-HEART study assessed the prevalence of abnormalities in cardiac structure and function in 803 PWH in the era of ART (15). The main findings of the study included LV dilatation in 10.1% of all PWH while 34 and 48% of patients had systolic and diastolic dysfunction, respectively. Severe forms of ventricular dysfunction were rare. In a large systematic review of 54 studies looking at cardiac dysfunction in ~125,382 PWH, there were 12,655 cases of cardiac dysfunction (16). The authors also found that there was a lower prevalence of LV systolic dysfunction in those studies reporting a higher use of HAART. LV systolic dysfunction was higher in the African region possibly reflecting lower access to HAART. These studies highlight the shift of HIVAC from a predominant systolic dysfunction to one of predominant diastolic dysfunction in the post ART era.

Echocardiography has been shown to be instrumental in early detection of cardiovascular disease and has also been helpful for researchers to diagnose differences between heart disease in PWH and in people without HIV. One study analyzed 1,195 participants from the Multicenter AIDS Cohort (MACS) study who underwent TTE exams demonstrating that men with HIV had a larger LV mass index and right ventricular size compared to controls (17). In addition to the structural differences found between groups, this study demonstrated that men with HIV had higher rates of diastolic dysfunction and RV dysfunction compared to men without HIV after adjusting for cardiovascular comorbidities. These cardiac abnormalities were also seen in virally suppressed men with HIV. The study however did not find any association between left ventricular ejection fraction (LVEF) and HIV seropositivity. Overall, the MACS study concluded that structural changes in HIV-positive patients may predispose them to heart failure with preserved ejection fraction.

Other studies evaluated LV systolic function by tissue Doppler strain echocardiography in PWH and participants without HIV. One study demonstrated that while LVEF in PWH was slightly lower than in patients who were uninfected, the LVEF values were still within normal limits. However, PWH had subtle cardiac dysfunction, as evidenced by reduced LV strain (18). Subclinical LV systolic dysfunction in HIV is further supported by other studies revealing no significant difference in conventional echocardiographic parameters including LVEF between PWH and people without HIV. However, the HIV population had significantly lower mean global longitudinal strain values (GLS). Furthermore, studies have shown that impaired GLS correlated with decreased CD4 counts (19, 20).

Right ventricular (RV) dysfunction is relatively common in PWH occurring largely in the setting of dilated cardiomyopathy or with varying degrees of HIV associated pulmonary hypertension (PH) (21). Isolated RV dysfunction has been documented in both the pre-ART and ART eras. RV dysfunction is associated with increased mortality in patients with PH and other forms of heart disease (22, 23). Therefore, understanding RV function in PWH is essential in determining the prognosis of patients with cardiac dysfunction and potentially guiding therapy. Prior to the widespread use of ART, smaller studies demonstrated isolated RV dilatation in PWH (13, 14, 24). In a more contemporary study, the prevalence of RV structural abnormalities and RV dysfunction was determined using echocardiography in 104 PWH on ART (25). RV dysfunction was common, but did not always correlate with elevated pulmonary artery systolic pressure (>35 mmHg) or LV systolic dysfunction, suggesting that RV dysfunction in PWH may occur independently of pulmonary hypertension or LV dysfunction.

The evidence highlights the value of echocardiography in the evaluation of myocardial function in PWH. Studies from the pre-HAART era demonstrate the pivotal role that echocardiography played in diagnosing systolic dysfunction in symptomatic patients often with advanced HIV disease. The same remains true today, and echocardiography remains an essential first line modality for assessing cardiac structure and function in PWH who present with heart failure symptoms. This non-invasive approach is important in characterizing biventricular function and cardiac structure as well as excluding other non-myocardial causes of symptoms. In the case of LV systolic dysfunction, evidence-based goal directed medical therapy should be considered per guideline recommendations (26). Although asymptomatic diastolic dysfunction is generally associated with an increased risk of symptomatic heart failure and death, screening echocardiography in asymptomatic PWH is not indicated although remains an area of active investigation. Rather the focus should be on aggressively managing comorbidities such as hypertension, obesity, or diabetes which may contribute to ventricular dysfunction.

## Cardiac Magnetic Resonance Imaging

Cardiac magnetic resonance (CMR) has been used to detect subtle and subclinical myocardial abnormalities, contributing to the current understanding of the pathogenesis of HIVAC. Untreated HIV infection is characterized by an immunodeficient state. In contrast, patients with well-treated HIV infection are in a state of immune activation. Several studies have shown a correlation between elevated inflammatory biomarkers and cardiovascular events in PWH (27). This observation has led to the postulation that inflammation may play an important role in the pathogenesis of HIV associated cardiovascular disease including myocardial dysfunction. At a cellular level, immune activation and chronic inflammation leads to deposition of collagen and myocardial fibrosis which is associated with an increased incidence of both systolic and diastolic dysfunction (28).

**Figure 1:**
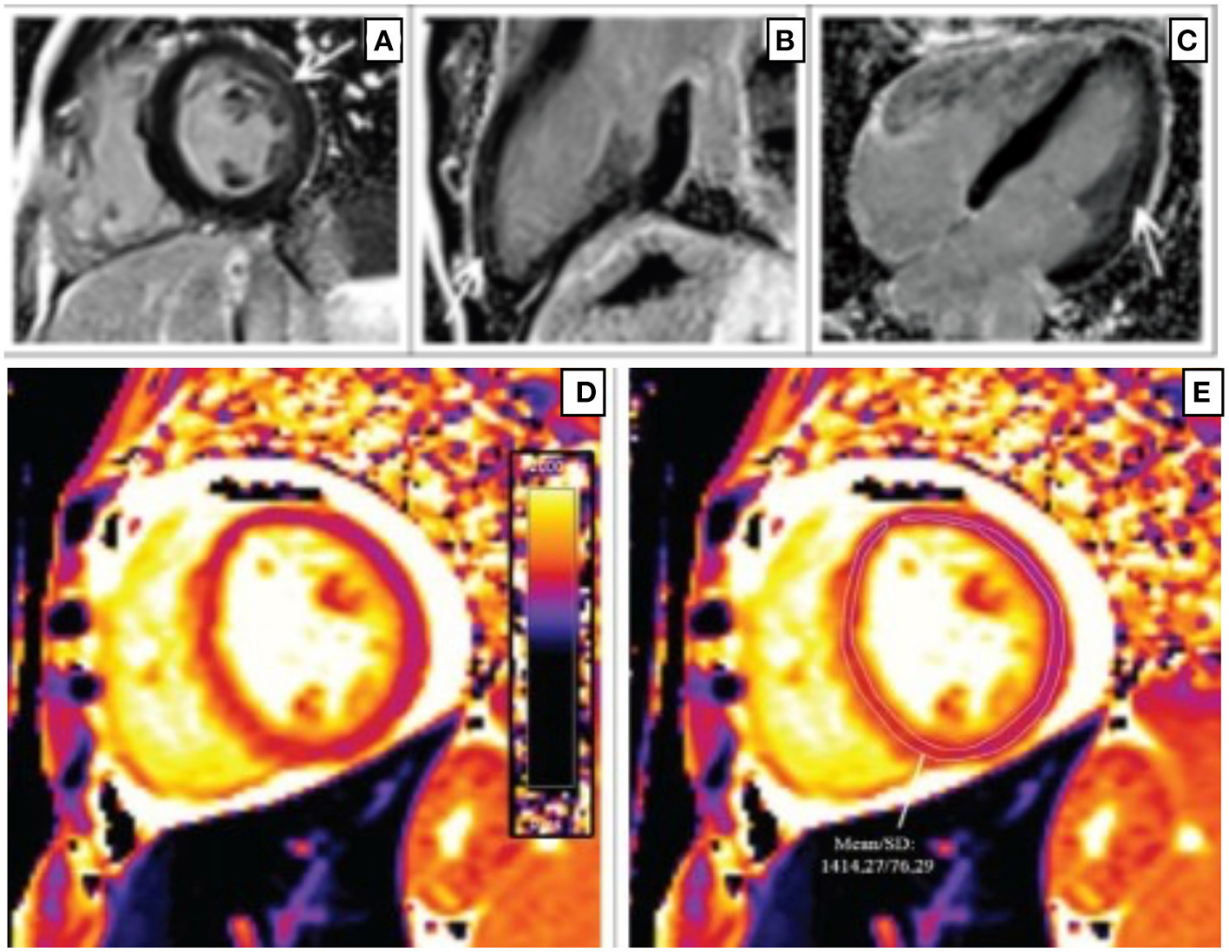
Short-axis **(A)**, 2-chamber **(B)**, and 4-chamber **(C)** late gadolinium phase-sensitive inversion recovery images show linear mid-wall enhancement in the lateral wall of the LV (white arrows) in a patient with HIV. T1 map at the level of the mid-LV **(D)** shows a markedly elevated myocardial T1 time of 1,414 m/s (normal 1,052 m/s ± 23 m/s at 3 T) **(E)** consistent with diffuse myocardial fibrosis (29).

CMR is a useful tool in assessing the role of inflammation and fibrosis in myocardial dysfunction in PWH. CMR can detect several components of inflammation, which include edema and fibrosis depending on the extent of cardiac involvement and stage of disease. Figure 1 shows CMR findings demonstrating fibrosis by late gadolinium enhancement (LGE) in a patient with HIV as well as markedly elevated myocardial T1 consistent with diffuse myocardial fibrosis (29).

Several studies have looked at the role of CMR in detecting subclinical myocardial dysfunction and fibrosis in PWH. In a study by Holloway et al., a total of 129 asymptomatic PWH on ART underwent CMR to assess cardiac function and myocardial fibrosis (30). Seventy-six percent of PWH were observed to have myocardial fibrosis predominantly in the basal inferolateral wall as compared to 13% of control subjects. In addition, peak myocardial systolic and diastolic strain were significantly lower in PWH. An extension of this study demonstrated that treated HIV infection was associated with chronic subclinical myocardial edema and pericardial effusions (31). Another study by Leutkens et al. studied PWH who were virally suppressed and underwent CMR. Compared to controls, the investigators found that PWH had lower LV strain values and ejection fraction, and higher myocardial inflammation by T2 weighted images. Myocardial fibrosis on LGE was also significantly elevated in PWH compared to controls (82% vs. 27%, *p* < 0.001) (32). A more recent study in South Africa demonstrated that PWH who were virally suppressed had greater myocardial fibrosis by extracellular volume (ECV) fraction compared with age and sex matched HIV negative controls. In addition, an elevated NT-proBNP level was associated with higher ECV (3.4%; 85% CI 1.3–5.5) (33). CMR has also played a role in tissue characterization of the RV in PWH, by demonstrating the presence of RV fibrosis by LGE (34). However, the clinical significance of these findings is yet unknown.

CMR plays an important role in the evaluation of HIV associated myocarditis. Before the introduction of ART, an early autopsy study demonstrated myocarditis in 52% of patients who died of AIDS (35). However, with the widespread use of ART, the incidence of HIV associated myocarditis has declined and the condition occurs almost exclusively in those with advanced immunosuppression. The gold standard for diagnosing myocarditis is endomyocardial biopsy however this techniques is invasive and can have low diagnostic yield (36). CMR on the other hand is a non-invasive imaging technique that permits detection of various stages of myocarditis with excellent diagnostic performance (37).

Measures of tissue characterization using CMR also have prognostic value for PWH. In a recent study, CMR was used to quantify ECV, reflective of myocardial inflammation or fibrosis, and showed that PWH have significantly higher ECV fraction when compared to people without HIV. The higher ECV value corresponded to more diffuse fibrosis, and studies have also shown a link between higher ECV values and increased adverse cardiovascular events. A prospective observational study evaluated the prognostic association between CMR measures and cardiovascular outcomes including heart failure in PWH on HAART. It was found that patients with diffuse myocardial fibrosis on CMR had a higher rate of cardiovascular events (38).

Lastly, novel non-contrast CMR techniques have been developed that may help in the work up of PWH who are prone to developing early vascular disease and microvascular dysfunction that may contribute to the pathogenesis of cardiomyopathy (39). Impaired coronary endothelial function (CEF) provides a promising early indication of coronary vascular disease in PWH. Abnormal CEF plays a critical role in the development, progression and clinical manifestations of coronary artery disease (CAD), independently predicts cardiovascular (CV) events, and is a target for medical interventions (40, 41). Using CMR combined with isometric handgrip exercise, an endothelial dependent stressor, studies in PWH revealed depressed CEF (measured by stress-induced change in coronary artery area and blood flow) compared to HIV negative matched adults (42).

## Cardiac Computed Tomography and Coronary Computed Tomography Angiography

Cardiac computed tomography (CT) and coronary CT Angiography (CCTA) have emerged as important imaging tools for evaluating both clinical and subclinical CAD in PWH. An analysis of the MACS cohort, a large cohort of HIV infected and matched uninfected men showed that after adjusting for known CVD risk factors, HIV infected men had an increased prevalence of non-calcified and mixed plaques compared to HIV uninfected men using CCTA (43). CCTA was also used to evaluate differences in the degree of coronary stenosis between the two groups. After adjustment for risk factors only a borderline association remained between HIV and increased degree of coronary artery stenosis.

Coronary artery calcification (CAC) is a highly specific marker of coronary atherosclerosis and is frequently employed to screen for CAD and assess risk for cardiovascular events (44). The MACS study showed that after adjustments for race, age, cohort and location, HIV infected men had a greater prevalence of CAC than HIV uninfected men (43). However, once adjustments for traditional CAD risk factors were made, the association between HIV and increased prevalence of CAC was borderline. Although coronary CT and CCTA are not indicated as screening tests in asymptomatic PWH, results from the MACS cohort and other studies emphasize the importance of evaluating and modifying traditional cardiovascular risk factors in this population.

PWH may present with symptomatic heart failure secondary to ischemic heart disease. In this setting CCTA is useful in the diagnostic workup of CAD. The utility of CT to establish the diagnosis of CAD as well as its value as a gatekeeper for invasive coronary angiography in patients with heart failure and LV systolic dysfunction was shown in a study of 93 patients with newly diagnosed heart failure of unknown etiology (45). If the CAC score was 0, the diagnosis of ischemic heart failure was ruled out; if the score was more than 0, CCTA was performed. Using this proposed algorithm, there was a sensitivity of 100%, with a specificity of 67% in the prediction of CAD.

## Nuclear Medicine Techniques

Nuclear medicine-based techniques have the ability to identify vascular inflammation and can permit the early detection of vascular disease in PWH (46). The degree of uptake of Fluorodeoxyglucose (FDG) in the carotid arteries and aorta detected by positron emission tomography-computed tomography (PET-CT) imaging techniques is useful in characterizing vascular inflammation, early atheroma and atherosclerotic plaques that are prone to rupture. One study reported a higher uptake of FDG in the carotid arteries and aorta of HIV infected individuals as compared to non-infected individuals (47).The use of myocardial perfusion imaging (MPI) with single-photon emission computerized tomography (SPECT) and PET have been evaluated in patients with HIV and found to have a high sensitivity and specificity in diagnosing CAD. These nuclear imaging techniques shed light on the etiology of cardiomyopathy by detecting cardiac stunning, presence of scar and assessing viability in patients with HIV thereby directing further management. In addition, the prevalence of RV dysfunction in a population of PWH was also investigated by radionuclide ventriculography in 95 patients (48). Although there was no significant LV dysfunction, a small but significant proportion of the cohort had modestly reduced RV systolic function defined as an ejection fraction <44%.

A summary of multimodality cardiovascular imaging techniques used for HIV-related heart disease is shown in Table 1.

**Table 1:** Clinical and investigational utility of cardiac imaging modalities in HIV associated cardiomyopathy.

	**ECHO**	**CMR**	**CT SCAN**	**Nuclear Imaging**
Chamber dimensions and volumes	^***^	^****^	^*^	^*^
Left ventricular systolic and diastolic function	^***^	^****^	–	^***^ (MUGA)
Mechanisms of ventricular dysfunction (ischemia vs. non ischemia)	^**^	^***^	^*^	^****^
Atherosclerosis	–	–	^***^	–
Myocardial tissue characterisation	^**^	^****^	–	–
Staging and monitoring disease progress	^***^	^****^	^**^	–
Limitations	Operator dependent and may be limited by acoustic windows Limited by geometric assumptions when assessing cardiac structure and function	Not widely available	Use of ionizing radiation, contrast (CTA)	Not widely available, patient preparation

*Echo, echocardiography; CMR, cardiac magnetic resonance; CT scan, computerized tomography; MUGA, multigated acquisition scan;*

* *poor*;

** *intermediate*,

*** *good*,

**** *excellent performance*.

## Discussion: Gaps in Knowledge and Future Directions

The introduction of HAART has led to a paradigm shift in the presentation of cardiovascular disease in PWH including that of HIVAC. Although the etiopathogenesis of HIVAC remains unclear, multimodality imaging has played a role in defining various putative mechanisms including inflammation and myocardial fibrosis. Multimodality imaging is also crucial in establishing a diagnosis of HIVAC in symptomatic patients. Although many studies have established prognosis in HIV patients with cardiomyopathy and systolic dysfunction, the prognostic significance as well as progression of diastolic dysfunction in HIVAC remains largely unknown and further long-term studies are needed. In addition, the mechanism and significance of right ventricular dysfunction especially in the absence of PH and LV dysfunction should be explored further. In summary, an approach tailored to the clinical presentation of the patient should be used to guide the use of cardiovascular imaging to identify myocardial dysfunction, investigate the possible underlying causes and facilitate appropriate preventive and evidence-based treatment for this condition.

## Author Contributions

EG, RR, and AH drafted the manuscript. MM, GS, EG, SB, SS, and BC reviewed and edited the manuscript. All authors contributed to the article and approved the submitted version.

## Funding

EG and AH received support from the Women as One Foundation. Further support was received from the National Institute of Health (R01HL147660) and the Johns Hopkins Center for AIDS Research (P30AI094189), Ruth L. Kirschstein Institutional National Research Service Award; T32HL007227-Pathophysiology of Myocardial Disease, the National Institute of Health.

## Conflict of Interest

The authors declare that the research was conducted in the absence of any commercial or financial relationships that could be construed as a potential conflict of interest.

## Publisher's Note

All claims expressed in this article are solely those of the authors and do not necessarily represent those of their affiliated organizations, or those of the publisher, the editors and the reviewers. Any product that may be evaluated in this article, or claim that may be made by its manufacturer, is not guaranteed or endorsed by the publisher.
